# Physical Exercise Attenuates Oxidative Stress and Morphofunctional Cerebellar Damages Induced by the Ethanol Binge Drinking Paradigm from Adolescence to Adulthood in Rats

**DOI:** 10.1155/2019/6802424

**Published:** 2019-02-18

**Authors:** Kátia Lamarão-Vieira, Dinair Pamplona-Santos, Priscila C. Nascimento, Márcio G. Corrêa, Leonardo O. Bittencourt, Savio M. dos Santos, Sabrina C. Cartágenes, Luanna Melo Pereira Fernandes, Marta C. Monteiro, Cristiane S. F. Maia, Rafael R. Lima

**Affiliations:** ^1^Laboratory of Functional and Structural Biology, Institute of Biological Sciences, Federal University of Pará (UFPA), Belém, PA, Brazil; ^2^Laboratory of Clinical Immunology and Oxidative Stress, Pharmacy Faculty, Institute of Health Science, Federal University of Pará, Belém, PA, Brazil; ^3^Laboratory of Inflammation and Behavior Pharmacology, Pharmacy Faculty, Institute of Health Science, Federal University of Pará (UFPA), Belém, PA, Brazil

## Abstract

Ethanol (EtOH) binge drinking is characterized by high EtOH intake during few hours followed by withdrawal. Protection strategies against the damages generated by this binge are poorly explored. Thus, this study is aimed at investigating the protective role of treadmill physical exercise (PE) on the damage caused after repeated cycles of binge-like EtOH exposure in the oxidative biochemistry, morphology, and cerebellar function of rats from adolescence to adulthood. For this, animals were divided into four groups: control group (sedentary animals with doses of distilled water), exercised group (exercised animals with doses of distilled water), EtOH group (sedentary animals with doses of 3 g/kg/day of EtOH, 20% *w*/*v*), and exercised+EtOH group (exercised animals with previous mentioned doses of EtOH). The PE occurred on a running treadmill for 5 days a week for 4 weeks, and all doses of EtOH were administered through intragastric gavage in four repeated cycles of EtOH in a binge-like manner. After the EtOH protocol and PE, animals were submitted to open field and beam walking tests. In sequence, the cerebellums were collected for the biochemical and morphological analyses. Biochemical changes were analyzed by measurement of Trolox equivalent antioxidant capacity (TEAC), reduced glutathione content measurements (GSH), and measurement of nitrite and lipid peroxidation (LPO). In morphological analyses, Purkinje cell density evaluation and immunohistochemistry evaluation were measured by antimyelin basic protein (MBP) and antisynaptophysin (SYP). The present findings demonstrate that the binge drinking protocol induced oxidative biochemistry misbalance, from the decrease of TEAC levels and higher LPO related to tissue damage and motor impairment. In addition, we have shown for the first time that treadmill physical exercise reduced tissue and functional alterations displayed by EtOH exposure.

## 1. Introduction

Ethanol (EtOH) is a global public health concern. Its abusive consumption involves about 3.3 million of deaths per year (5.9% of all world deaths) and 5.1% of morbidity in the world [1]. From the pharmacological point of view, EtOH is a dose-dependent psychotropic drug that when consumed in large proportions increases the risk of morbidity and/or mortality [2, 3].

Over the years, the pattern of EtOH consumption has shown significant changes, both in quantity and frequency [4]. Binge drinking has been considered a toxic and dangerous practice because it is an intense consumption (≤0.08 g/dL of EtOH in the blood) in a single session, followed by abstinence, which causes changes in the central nervous system (CNS) [5, 6]. Nowadays, the most emerging pattern of EtOH intake by adolescents and young people is the binge drinking, characterized by high EtOH intake during few hours followed by withdrawal [6, 7].

In face of the high prevalence of EtOH consumption, investigating modifiable factors for CNS damage becomes an important aim for neuroprotective strategies. Physical exercise (PE) has been shown to modulate neural functions due to its neuronal loss preventions after several brain damages. Also, it acts on behavioral improvement and structural alteration in the brain [8], promotes plasticity [9–11], modulates the oxidative biochemistry and synaptic transmission [12, 13], increases brain-derived neurotrophic factors (BDNF), and stimulates angiogenic processes [10].

Considering the epidemiological relevance of EtOH and the possible injuries to the cerebellum, organ that plays an important role in the control of the sensorimotor system, motor coordination, and motor cognition [14–16], this study is aimed at investigating the action of moderate treadmill PE as a possible neuroprotective strategy to avoid damages displayed by the EtOH binge drinking pattern over the cerebellum, as well as the motor functions associated to this brain structure of rats.

## 2. Materials and Methods

### 2.1. Animals and Experimental Design

Forty male Wistar rats, weighing 100-150 g with the age of 30 days, were used in this investigation. The animals were maintained in collective cages (4 animals each) with water and food *ad libitum* and kept in a climate-controlled room (25°C) with a 12 : 12 h light/dark cycle (lights on 7:00 AM). All procedures were previously approved by ethics committee on animal experimentation by Federal University of Pará, under the protocol CEPAE-UFPA: 227-14, and following the NIH Guide for the Care and Use of Laboratory Animals [17].

**Figure 1:**
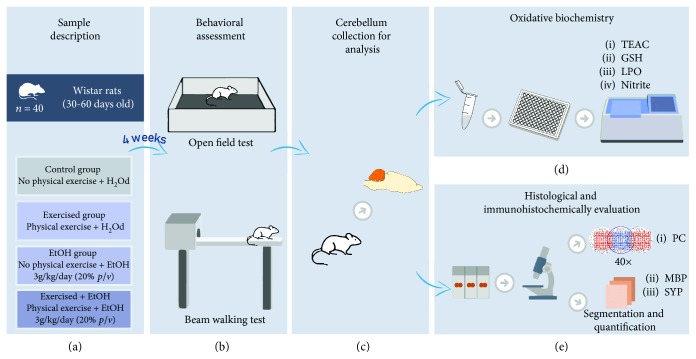
Sample description and experimental stages. (a) Description of the sample and division of experimental groups of treadmill physical exercise and ethanol (EtOH) or distilled water (H_2_Od) administration. (b) The behavioral assays: open field and beam walking tests. (c) Cerebellum collection for analyses. (d) Oxidative balance analyses through Trolox equivalent antioxidant capacity (TEAC), reduced glutathione (GSH), lipid peroxidation (LPO), and nitrite levels (nitrite). (e) Histological analysis by quantification of Purkinje cells (PC) and immunohistochemically evaluation through anti-synaptophysin (SYP) and anti-myelin basic protein (MBP).

Experimental animals were randomly distributed into four groups: control group (sedentary animals that received distilled water by gavage, *n* = 10), exercised group (exercised animals that received distilled water by gavage, *n* = 10), EtOH group (sedentary animals that received 3 g/kg/day of EtOH, 20% (*w*/*v*) by gavage, *n* = 10), and exercised+EtOH group (exercised animals with EtOH doses previously mentioned, *n* = 10). The experimental design is summarized in Figure 1.

**Figure 2:**
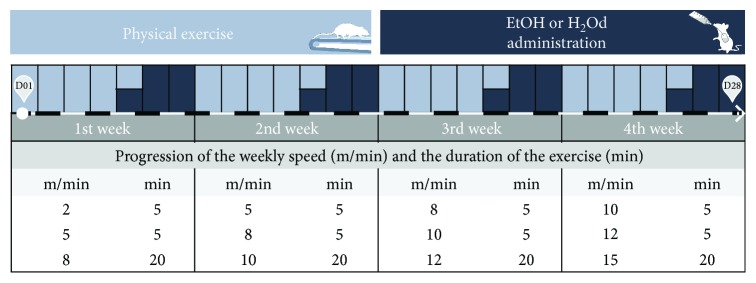
Experimental design of the treadmill physical exercise protocol and ethanol (EtOH) or distilled water (H_2_Od) administration by intragastric gavage since day 1 (D01) to day 28 (D28). The physical exercise protocol was adapted from Arida et al. [18].

The PE protocol was adapted from Arida et al. [18], in which the animals were subjected to 5 consecutive training days per week for 4 weeks on the treadmill without inclination degree (MasterOne). The physical exercise scale is described in Figure 2. Five days after treadmill PE, one cycle of binge-like EtOH treatment was performed. This cycle involves a single EtOH administration per day by oral gavage, for 3 consecutive days (3 days followed by 4 days off). Control subjects received only distilled water, in the same paradigm [5, 19, 20]. The cycle of treadmill PE and binge-like EtOH exposure lasted for 4 weeks, and the animals were weekly weighted for dose adjustment.

### 2.2. Behavioral Assay

Twenty-four hours after the last protocol day of EtOH intake and treadmill PE, animals were conducted to the assay room for motor behavioral tests. Open field and beam walking tests were conducted between 11:00 AM and 6:00 PM in a sound-attenuated room under low-intensity light (12 lux), where the rats had been habituated for at least 2 h before beginning the tests.

#### 2.2.1. Open Field Test

The analyses of spontaneous locomotion and vertical exploratory activity were assessed by open field protocol [21]. Animals were placed on the arena (100 × 100 × 40 cm), wherein the floor is divided into 25 equal quadrants (20 × 20 cm). Firstly, each animal is placed in the center of the floor and observed for five minutes. The number of total intersections was analyzed by the ANY-maze™ software (Stoelting, USA), and the number of standing position (rearing) was analyzed manually.

#### 2.2.2. Beam Walking Test

After open field assay, animals were submitted to beam walking test. In this experiment, motor coordination and balance were assessed based on animals' ability to traverse a graded series of narrow beams to reach an enclosed safety platform. The apparatus consists of wooden beams (100 cm length) suspended 50 cm from the floor, which allows the rat to access a secure platform (closed box of 20 × 20 cm). Briefly, animals were acclimated in the beams of a higher cross-sectional area, and a limit of 120 s was fixed to reach the box. In order to intensify the task difficulty, animals were submitted to two test sessions (cut-off 60 s each) on each beam of square cross section (12 mm and 5 mm), respectively. The latency to reach the closed box was measured in seconds, and the number of foot slips was registered considering one or both hind limbs slipped from the beam [22, 23].

After behavioral tests, five animals per group were euthanized by cervical dislocation and the cerebellums were immediately removed for biochemical assay. The other 5 animals from each group were perfused, and the cerebellums were destined for histological evaluations.

### 2.3. Oxidative Biochemistry Analyses

After cerebellum collection, the dissected tissue was cleaned in saline solution, frozen in liquid nitrogen, and subsequently stored at −80°C. Then, the tissue was thawed and resuspended in Tris-HCl 20 mM, pH 7.4 at 4°C, and sonically disaggregated. The preparation of the tissue for oxidative biochemistry analysis has been described in detail in our previous studies [22, 24].

#### 2.3.1. Measurement of Trolox Equivalent Antioxidant Capacity (TEAC)

For TEAC level analyses, we used the method described by Rufino et al. [25] and the method adapted from our previous study [26]. Briefly, the 2,2′-azino-bis(3-ethylbenzothiazoline)-6-sulfonic acid (ABTS; 7 mM) was incubated by adding potassium persulfate (2.45 mM) at room temperature during 16 h to produce ABTS+ radical. The work solution was prepared from ABTS+ radical in phosphate basic saline (PBS) solution (pH 7.2) until absorbance of 0.7 ± 0.02 at 734 nm. Subsequently, 35 *μ*L of this solution or Trolox standards (standard curve) was added to 2970 *μ*L of ABTS solution, and absorbance was acquired after 5 minutes. Absorbances were measured in triplicate and calculated following a standard curve with Trolox [27] standard concentrations. Total antioxidant capacity was expressed in *μ*mol/L.

#### 2.3.2. Reduced Glutathione Content Measurements (GSH)

Determination of the GSH levels was based on the ability of GSH to reduce 5,5-dithiobis-2-nitrobenzoic acid (DTNB) to nitrobenzoic acid (TNB), which was quantified by spectrophotometry at 412 nm. Thus, the methodology described by Ellman [28] was adapted for this determination. Initially, an aliquot (20 *μ*L) from supernatant was added in a tube containing distilled water (20 *μ*L) and PBS solution pH 8.0 (3 mL) to carry out the first measurement. Afterwards, 5,5′-dithiobis (2-nitrobenzoic acid) (DTNB; 0.47mMol) was added to the solution, and another measurement was carried out after 3 minutes [26, 29]. The GSH concentration was expressed as *μ*g/mL.

#### 2.3.3. Determination of Lipid Peroxidation (LPO)

Lipid peroxidation was estimated as the levels of malondialdehyde (MDA) and 4-hydroxyalkenes (4HDA) as earlier detailed by Esterbauer and Cheeseman [30]. As previously described in our studies [5, 31], an aliquot of the supernatant was processed as described by the Bioxytech LPO-568 kit (Cayman Chemical). This kit is a chromogenic reagent that reacts with MDA at 45°C. The absorbance measurement on spectrophotometers was performed at 586 m wavelength.

#### 2.3.4. Nitrite Level Quantification

For quantification of nitrite, we used the protocol described by Green et al. [32], which consists of using an aliquot of crude homogenate that was centrifuged at 21,000 for 20 min at 4°C and the supernatant to analyze nitrite levels. Briefly, the samples were incubated at room temperature for 20 min with the Griess reagent (0.1% naphthylethylenediamine and 1% sulfanilamide in 5% phosphoric acid—1 : 1). The absorbance was measured at 550 nm by a spectrophotometer and compared to the standard sodium nitrite solutions.

#### 2.3.5. Protein Concentration Assay

The measurement of protein content in the supernatants (20 *μ*L) was performed as described by Bradford [33] in order to correct the values of LPO and nitrite by protein concentration. Thus, results of LPO and nitrite levels were expressed in percentages of control groups.

### 2.4. Histological Evaluation

After behavioral assessment, 5 animals of each group were used for tissue analysis. The animals were deeply anesthetized with ketamine hydrochloride (90 mg/kg) and xylazine hydrochloride (10 mg/kg) solution and transcardially perfused with heparinized 0.1 M phosphate-buffered saline (PBS) followed by 4% paraformaldehyde. The cerebellums were removed from the cranial cavity and postfixed for 4 hours in Bouin solution. Then, the cerebellums were dehydrated in increasing EtOH solutions, diaphanized in xylol, and embedded in paraplast (McCormick®). Sections of 5 *μ*m thickness were obtained by coronal cuts in microtome and then put on microscopy slides.

For analysis of Purkinje cells, tissue sections were stained with hematoxylin and eosin (HE) and for immunohistochemistry analysis the sections of the same animal cerebellum were mounted on 3-aminopropyltriethoxysilane-coated (Sigma®) microscopy slides. The number of Purkinje cells was evaluated by using a square 0.25 mm wide grid in the eyepiece of the microscope for quantitative assessments. This grid corresponds to an area of 0.0625 mm^2^. At least 3 fields in the cerebellum per section and 3 sections per animal of each group were analyzed, adapted from Lima et al. [34] for the cerebellum.

#### 2.4.1. Immunohistochemistry

Myelin impairment was evaluated using an antibody against rat myelin basic protein (MBP), an important component of the compact myelin. In addition, we used antisynaptophysin (SYP) for immunostaining of neurosecretory vesicles.

All immunohistochemistry procedures were conducted according to the previously published studies [34–36]. Briefly, sections were dewaxed in xylene, hydrated in decreasing ethanol solutions (absolute 2, absolute 1, 90%, 80%, 70%), and rinsed in 0.1 M PBS for 5 min. Antigen recovery was performed with citrate buffer solution (pH 6.0), previously heated to 60°C, for 20 min. After that, sections were further allowed to cool for about 20 min and incubated in 1% hydrogen peroxide solution (H_2_O_2_) in methanol for 20 min for the inhibition of endogenous peroxidase activity. Then, sections were rinsed three times in 0.1 M PBS/Tween (Sigma®) solution for 5 min and incubated with 10% normal horse serum and 3% bovine serum albumin (BSA, Sigma®) in PBS for 1 h. Without further rinsing, sections were then incubated overnight with the primary antibody in PBS: anti-MBP (1 : 100, Chemicon®) and anti-MBP (1 : 100, Serotec®), rinsed in PBS/Tween solution for 5 min (three times), and incubated with biotinylated horse anti-mouse (1 : 100) secondary antibody (Vector Laboratories®), for 2 h. Sections were rinsed again for 5 min (three times) and incubated in avidin-biotin-peroxidase complex (ABC Kit, Vector Laboratories®) for 2 h. Sections were rinsed three times (five minutes each) in 0.1 M phosphate-buffered saline and revealed with 3,3′-diaminobenzidine (DAB). After DAB reaction, sections were rinsed two times in 0.1 M PBS, counterstained by Mayer's hematoxylin, dehydrated using alcohols and xylene, and coverslipped with Entellan (Merck®).

For quantitative analysis of MBP and synaptophysin immunostaining, photomicrographs were acquired by an Axioscope microscope (Carl Zeiss, Germany) equipped with an AxioCam HRC CCD Color Camera (Carl Zeiss) with the same magnification of 40x. They were segmented by “deconvolution color plugin” (Gabriel Landini, http://www.dentistry.bham.ac.uk/landinig/software/software.html) using the ImageJ software (NIMH, NIH, Bethesda, MD, USA, https://imagej.nih.gov/ij/). Afterwards, the area fraction values (%) of DAB staining were measured in the sections [34, 37, 38]. The photomicrographs were acquired in the region of the second cerebellar leaf of 5 sections per animal, 3 micrographs per section (5 animals per group). The values obtained were expressed as mean ± standard error.

### 2.5. Statistical Analyses

After data collection, all the results were tabulated and analyzed by GraphPad Prism 7.0 software (GraphPad Software Inc., La Jolla, CA, USA); the data distribution was tested by the Shapiro-Wilk method for verification of normality. The weight curve was evaluated with two-way ANOVA followed by the Tukey post hoc test. Oxidative biochemistry, Purkinje cell analysis, and behavioral assays were performed by one-way ANOVA and the Tukey post hoc test, except for immunohistochemical labeling, in which we use the nonparametric Mann-Whitney test, due to fraction area analysis. The results were expressed in mean ± standard error of the mean (SEM), and values of *p* ≤ 0.05 were considered statistically significant.

## 3. Results

### 3.1. Repeated Cycles of EtOH in a Binge-Like Manner and Treadmill Physical Exercise Did Not Interfere in Animals' Weight Gain

**Figure 3:**
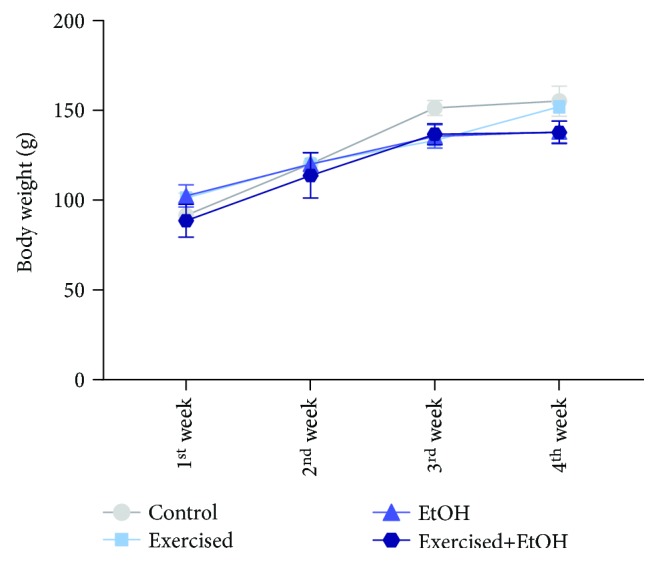
Effects of repeated cycles of treadmill physical exercise and exposure to binge-like ethanol, during 4 weeks on body weight gain (g) from adolescence through adulthood in Wistar rats. Results are expressed as mean ± standard error of mean. Two-way ANOVA and Tukey's post hoc test, *p* > 0.05.

After repeated cycles of treadmill physical exercise and binge-like EtOH during 4 weeks, we did not observe alteration on animals' weight (*p* = 0.538; Figure 3). At the end of the experiment, the animals had no difference in mean weight (control group: 129.6 ± 14.9, exercised group: 126.7 ± 10.69, EtOH group: 124 ± 8.232, and exercised+EtOH group: 119.3 ± 11.62).

### 3.2. Regular Treadmill Physical Exercise Minimized Oxidative Stress Induced by Four Cycles of Binge Drinking in the Cerebellum of Rats

**Figure 4:**
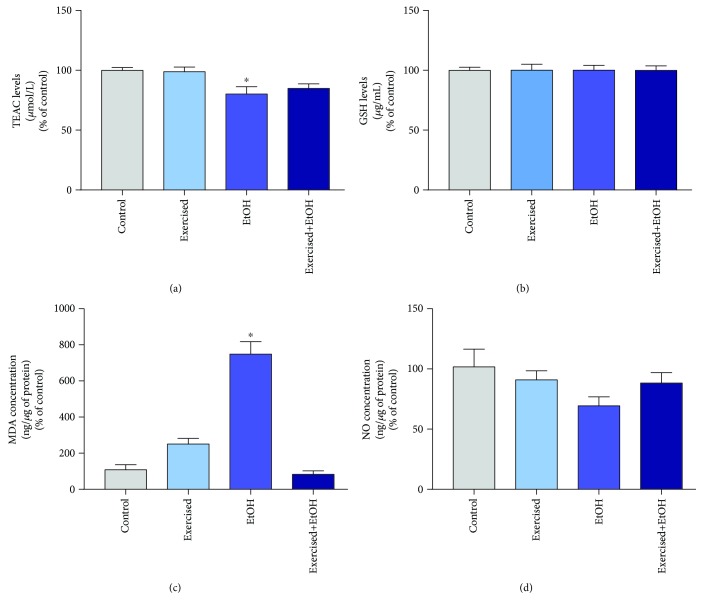
Effects of repeated cycles of treadmill physical exercise and exposure to binge-like ethanol, during 4 weeks, on oxidative balance in the cerebellum of 60-day-old Wistar rats. (a) TEAC levels, (b) GSH levels, (c) percentages of milligram malondialdehyde per protein in relation to the control group, and (d) percentages of nitrite per milligram of protein in relation to the control group. Results are expressed as mean ± standard error of mean. One-way ANOVA and Tukey's *post hoc* test, *p* < 0.05. ^∗^Statistical difference in relation to the other groups.

The binge-like EtOH exposure decreased TEAC levels (EtOH group: 80.13 ± 6.16; Figure 4(a)) in the cerebellum of rats compared to the control animals (99.88 ± 2.445; *p* = 0.015). Such imbalance of oxidative biochemistry was avoided by physical exercise (exercised+EtOH group: 84.87; *p* = 0.863; Figure 4(a)). In addition, as observed in Figure 4(b), exposure to EtOH did not change the oxidative parameters related to GSH levels (*p* > 0.999) in the studied groups.

On the other hand, prooxidant factors were found to be statistically increased. The exposure to binge drinking of EtOH for 4 weeks was able to modulate oxidative biochemistry by increasing LPO levels (EtOH group: 748.3 ± 69.62%) in the cerebellum of the animals, showing statistical difference compared to the control group (100.00 ± 28.05%; *p* < 0.0001) as shown in Figure 4(c). In contrast, the physical exercise reduced the lipid peroxidation in the cerebellum, reflecting neuroprotection against EtOH disturbance (exercised+EtOH group: 83.02 ± 19.25% versus the control group; *p* = 0.977). No difference in nitrite concentration among groups was detected (*p* = 0.178; Figure 4(d)).

### 3.3. Physical Exercise Reduced Cerebellar Tissue Damage Induced by Binge-Like EtOH Exposure for 4 Weeks in Rats

**Figure 5:**
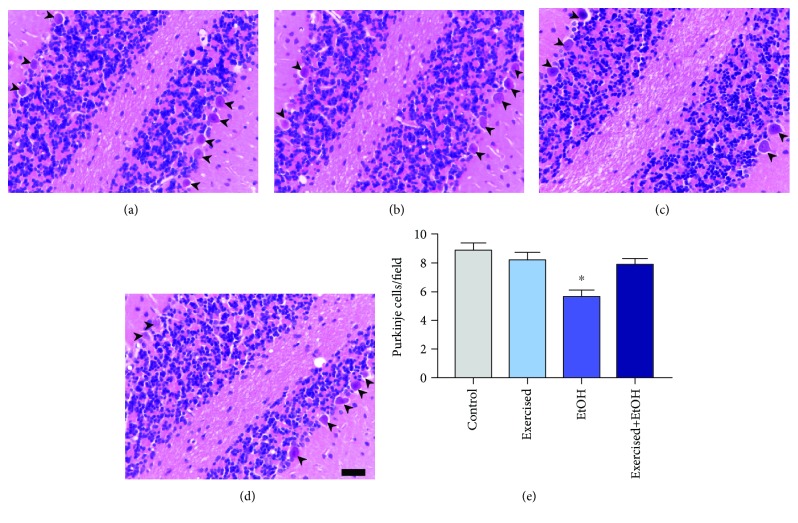
Effects of repeated cycles of treadmill physical exercise and exposure to binge-like ethanol, during 4 weeks, on Purkinje cells in the cerebellum of 60-day-old Wistar rats. Representative photomicrographs of the (a) control group, (b) exercised group, (c) EtOH group, and (d) exercised+EtOH. Arrowheads indicate Purkinje cells. Results are expressed as mean ± standard error of the number of cells counted per field (e). One-way ANOVA and Tukey's post hoc test, *p* < 0.05. ^∗^Statistical difference in relation to the other groups. Scale bar 20 *μ*m.

In order to analyze whether binge-like EtOH exposure can induce alterations in cerebellar tissue morphology, we performed HE staining. Our results show that binge-like EtOH exposure reduces Purkinje cell population in the rats (EtOH: 5.667 ± 0.441) when compared to the control group (control group: 8.875 ± 0.5154; *p* = 0.0002; Figure 5). On the other hand, the physical exercise avoided the damage, once the trained groups did not show significant difference when compared to the control group (exercised group: 8.222 ± 0.4938; exercised+EtOH group: 7.875 ± 0.4407; *p* > 0.481; Figure 5).

**Figure 6:**
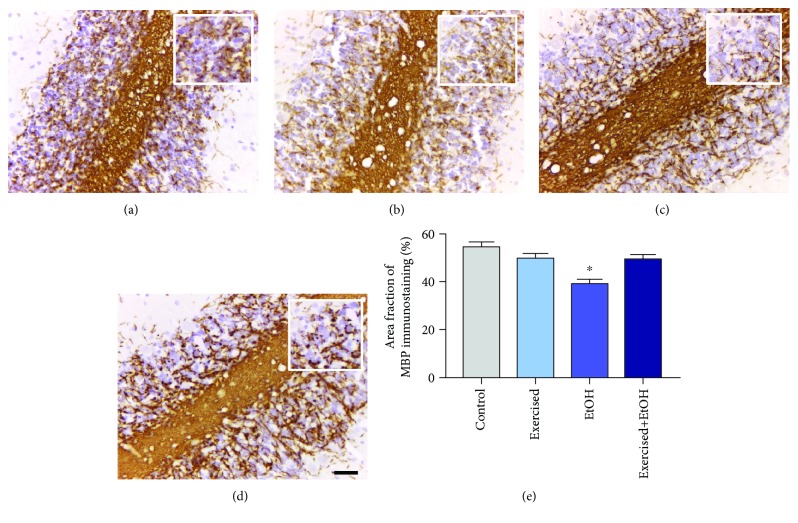
Myelin basic protein (MBP) immunostaining in the cerebellum of 60-day-old Wistar rats exposed to repeated cycles of treadmill physical exercise and exposed to binge-like ethanol, during 4 weeks. Representative photomicrographs of the (a) control group, (b) exercised group, (c) EtOH group, and (d) exercised+EtOH. Results are expressed as mean ± standard error of area fraction percentage of immunostaining (e). One-way ANOVA and Tukey's post hoc test, *p* < 0.05. ^∗^Statistical difference in relation to the other groups. Scale bar 20 *μ*m.

In addition to the morphological changes caused by exposure to EtOH in a binge-like manner, we observed that the proposed pattern was able to decrease the area fraction of MBP immunostaining in the group exposed only to EtOH (EtOH group: 39.54 ± 1.822; *p* < 0.0001) compared to the other groups. Interestingly, the treadmill PE paradigm group reduced the loss of the MBP fraction area (exercised+EtOH group: 49.88 ± 1.817; control group: 54.91 ± 2.085; *p* = 0.243), as seen in Figure 6, indicating a possible neuroprotection of the cerebellar damage of rats.

**Figure 7:**
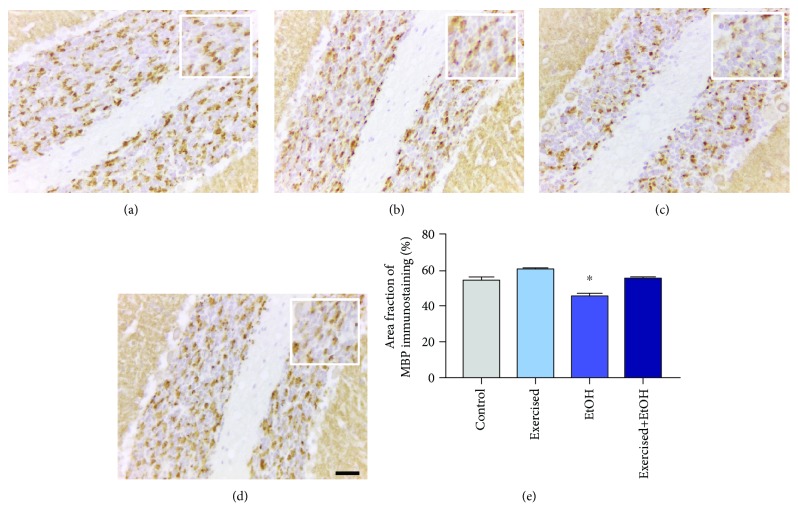
Synaptophysin (SYP) immunostaining in the cerebellum of adult Wistar rats exposed to repeated cycles of treadmill physical exercise and exposure to binge-like ethanol during 4 weeks. Representative photomicrographs of the (a) control group, (b) exercised group, (c) EtOH group, and (d) exercised+EtOH. Results are expressed as mean ± standard error of area fraction percentage of immunostaining (e). One-way ANOVA and Tukey's post hoc test, *p* < 0.05. ^∗^Statistical difference in relation to the other groups. Scale bar 20 *μ*m.

In addition, we also demonstrated a remarkable decrease in the area fraction of synaptophysin immunostaining (*p* < 0.0001), indicating that our model of exposure to EtOH causes damage to synaptic vesicles but can be minimized by treadmill physical exercise (control group: 54.69 ± 1.333; exercised+EtOH: 55.45 ± 0.8258; *p* = 0.956; Figure 7).

### 3.4. Cerebellum Biochemical and Histological Alterations Reflected in Poor Performance on Spontaneous Locomotor Activity Assays of Rats after Binge-Like EtOH Exposure for 4 Weeks

**Figure 8:**
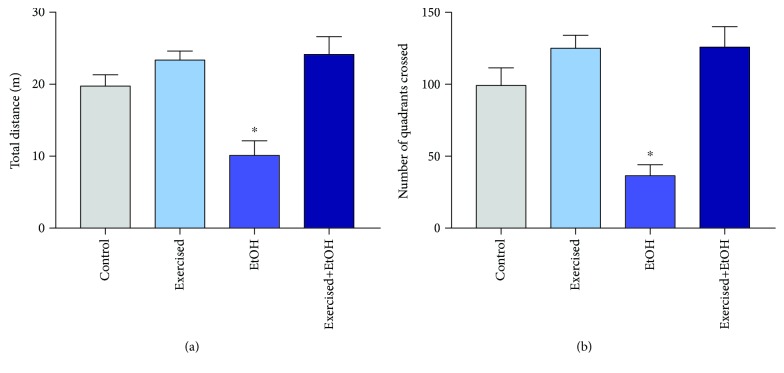
Effects of repeated cycles of treadmill physical exercise and binge-like ethanol, during 4 weeks, on spontaneous locomotor of 60-day-old Wistar rats. (a) Total distance (m) and (b) number of quadrants crossed in the open field test. One-way ANOVA and Tukey's post hoc test, *p* < 0.05. ^∗^Statistical difference in relation to the other groups.

Binge-like EtOH exposure for 4 weeks in rats induced spontaneous motor behavior deficits in behavioral tasks. However, the treadmill physical exercise triggered the performance of the spontaneous locomotor activity, both in the total distance travelled (control group: 19.74 ± 1.562; EtOH group: 10.11 ± 2.021; *p* = 0.005; Figure 8(a)) and in the number of crossed quadrants (control group = 99.22 ± 12.17; EtOH group: 36.44 ± 7.658; *p* = 0.0014; Figure 8(b)) on the arena that was impaired by the EtOH protocol (*p* < 0.0001), as observed in Figure 8.

**Figure 9:**
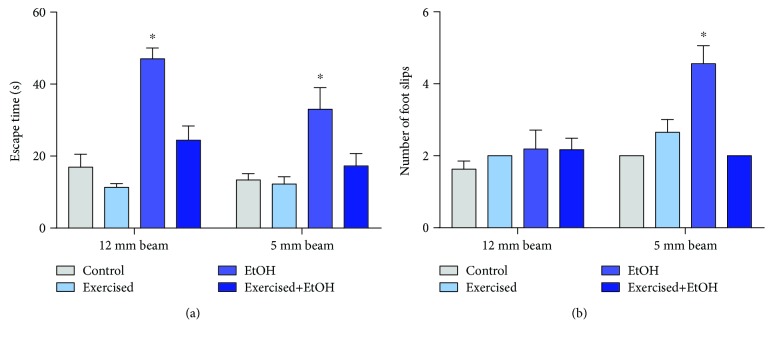
Effects of repeated cycles of physical exercise and binge-like ethanol, during 4 weeks, on fine motor coordination and balance of 60-day-old Wistar rats. The results are expressed as mean ± standard error of (a) latency (s) and (b) failure numbers in 12 mm and 5 mm beams of the beam walking test. One-way ANOVA and Tukey's post hoc test, *p* < 0.05. ^∗^Statistical difference in relation to the other groups.

In the motor coordinating and balance test, subjects exposed to EtOH administration presented poor performance on the beam walking test, increasing the latency to reach the safe box, on both 12 mm and 5 mm beams (EtOH group: 12 mm: 47 ± 3, 5 mm = 33 ± 6; control group: 12 mm: 16.91 ± 3.596, *p* < 0.001; 5 mm: 13.38 ± 1.749, *p* < 0.001; Figure 9(a)). In addition, the EtOH group increased the number of foot slips during the challenges with the smaller beam compared to other groups (5 mm: control group: 2 ± 0.0; exercised group: 2.65 ± 0.3578; EtOH group: 4.556 ± 0.5031; exercised+EtOH group: 2 ± 0; *p* = 0.005; Figure 9(b)). However, the treadmill physical exercise improved the animal performance, reducing the latency to cross the beams, as well as the foot slips, restoring the accomplishment of the control individuals.

## 4. Discussion

In this study, we demonstrated that EtOH exposure by means of 4 binge episodes from adolescence through adulthood is able to promote biochemical, tissue, and functional changes in the cerebellum of rats, which can be minimized due to the neuroprotection provided by concomitant treadmill physical exercise. The present findings demonstrate that the proposed binge ethanol drinking protocol induces oxidative biochemistry disbalance characterized by a decrease in TEAC levels and high lipid peroxidation, which can trigger tissue and behavioral damages in rats. In addition, we showed for the first time in literature that such damages in the cerebellum of adult rats promoted by binge drinking could be minimized by treadmill physical exercise.

Brain control of movements is performed by several regions of the CNS; among them, the cerebral cortex is responsible for motor planning and programming, in addition to command both from the spinal cord and brainstem that modulate reflexes and coarse movements [15]. A sensitive and accurate control system is performed by the cerebellum and basal ganglia [16, 39]. We have chosen the cerebellum as the investigation area since this structure modulates motor pathways and plans and coordinates movements, as well as motor learning [40].

In this context of motor control, the cerebellum is considered as the CNS region that integrates movement and posture, so as to control distance and range of motion [15]. In order to modulate movement, the afferent and efferent fibers of the cerebellar circuit communicate with the various regions of the nervous system (NS). The climbing and mossy fibers form excitatory synapses with deep cerebellar nuclei and Purkinje cells, directly or indirectly [16]. In our study, we evaluated the density of MBP, the synaptic communication pattern, and the preservation of Purkinje neurons in the cerebellar leaves.

Purkinje neurons were chosen to analyze the damage caused by ethanol, since they figure as the main elements of cerebellar circuitry; these cells are of fundamental importance in sensorimotor calibration due to their activity change during motor tasks in order to promote performance excellence [16]. In addition, Purkinje neurons can predict sensorimotor activities, transfer plasticity from cerebellar cortex to deep nuclei, and directly contribute to the motor command [40]. In our study, we found a decrease of Purkinje neuron density in animals exposed to ethanol, a fact that may have contributed to the functional damages observed.

Furthermore, we found an immunolabeling reduction of the synapse-related protein synaptophysin after 4 weeks of binge drinking; however, it is important to emphasize that cerebellar neurons are able to engage compensatory mechanisms to maintain neurotransmission at normal levels [15]. In addition, EtOH has been shown to promote alteration in other cell structures such as cytoplasmic organelles, nucleus, and cytoskeleton as well as changes in neuronal synaptic contacts in cell cultures of the cerebral cortex of rats [41].

Besides reducing Purkinje neuron density and synaptic communication, a binge EtOH pattern was able to damage the myelin sheath. It is known that damage to these structures can cause deficits in electrical impulse conduction during action potential in myelinated neurons, which are the main responsible for motor response [42]. We believe that these data gather evidence to support our hypothesis that binge-like ethanol exposure may trigger cerebellar damages, which can be suggested as responsible elements for motor impairment found in our investigation.

Deficiency in thiamine synthesis, retinoic acid modulation, and neuroinflammation can be identified as responsible elements for CNS damages caused by EtOH, whereas oxidative stress is a strong mechanism also associated with damage as described by the previous studies [5, 43, 44]. Oxidative stress results from an imbalance between production and removal of reactive oxygen species and causes severe cellular damage that may lead—after following hierarchical damages—to behavioral alterations [44].

Although the literature indicates oxidative stress as one of the main mechanisms of CNS damage caused by EtOH [45–47], our research is the first to describe the oxidative changes in the cerebellum of rats triggered by the binge pattern and EtOH consumption from adolescence through adulthood in rats. We have shown that even with 4 binge episodes during this period, there is a decrease in total antioxidant capacity (TEAC) and an increase in lipid peroxidation (MDA) levels on the cerebellum; this was previously reported by other studies but through chronic consumption [44, 45, 48]. Even though neural damage mechanisms have not yet been fully understood, oxidative stress is considered one of the most relevant [46, 49]. EtOH intoxication due to acute, chronic, or binge exposure leads to oxidative stress as a result of an imbalance between free radicals (FRs) and antioxidants, followed by the formation of reactive species (RSs) and lipid peroxidation [43, 44]. Recently, our group also showed that ethanol exposure led to oxidative stress with increased MDA levels, possibly reflecting on the CNS, which led to a profile of psychiatric disorders or cognitive impairment [50]. Thus, this increase in the levels of MDA caused by ethanol consumption may be associated with a higher CAT activity induced by the ethanol metabolic process, which generates excess H_2_O_2_ and hydroxyl radicals (OH^•^), one of the most potent molecules leading to oxidation and damage to biomolecules [51]. Therefore, we confirm here that the toxicity of ethanol during its metabolism leads to a marked oxidative stress due to the powerful oxidizing molecules in the body.

When we evaluated the motor activity of our exposure model (binge), the results indicated that EtOH promoted changes in exploratory capacity, since the exposed animals walked through smaller number of quadrants and shorter total distance. Moreover, at the beam walking test, animals that consumed alcohol presented worse motor performance characterized by higher number of falls and less ability for activities that required motor dexterity. These results corroborate those recently found in another study from our group, which demonstrated that binge episodes during adolescence impact motor performance [50, 52], as well as affect other brain areas, inducing an anxiogenic behavior and influencing short-term recognition memory [6]. We believe that ethanol consumption increased cerebral blood flow by increasing the metabolic oxygen demand, so that brain regions related to motor activity and central command network are affected. Thus, increased oxygen consumption results in increased production of a variety of reactive oxygen species (ROS), such as superoxide anion, OH^•^, nitric oxide (NO), and singlet oxygen due to oxidation leakage of electrons related to the mitochondrial transport chain phosphorylation [50, 53]. Thus, ROS can cause oxidative damage to cellular components by impairing cellular energy and modulating signaling pathways (“redox signaling”) that lead to several acute and chronic changes in the cellular environment, especially in the CNS [54]. Thereby, overall, the decreased antioxidant levels and increase of ROS in the cerebellum may be responsible for cellular damage, including in Purkinje neurons, which resulted in impairment in motor activity, anxiogenic behavior, and a short-term recognition memory failure.

On the other hand, there are some strategies to reverse, minimize, or protect the CNS from alterations caused by oxidative stress [55–57]. Among neuroprotection strategies, exercise is a subcategory of physical activity that involves several exercise methods such as volume, frequency, and intensity. Moreover, muscular strengthening exercise mostly relies on anaerobic energy sources [58]. On the contrary, exercises of aerobic resistance generally use mainly aerobic energy sources, so they were chosen for this research [56]. Thus, regular moderate exercise is an essential factor for maintaining good health and helps in the prevention of chronic diseases, including neurodegeneration [59–61]. In this regard, several studies reported that moderate regular exercise beneficially stimulates brain function by playing an important preventive and therapeutic role in cases of stroke and in Alzheimer's and Parkinson's diseases [62–65]. However, these beneficial effects are broad, including the stimulation of neurogenesis via neurotrophic factors, increased capillarization, decreased oxidative damage, and increased proteolytic degradation by proteasome and neprilysin, among others [66].

Although muscular strengthening exercises present some relevance regarding the improvement of CNS-related parameters such as motor skills, balance, and coordination [67], we have chosen aerobic exercise as a possible oxidative stress modulator in the cerebellum due to its ability to modulate oxidative stress in other tissues, such as blood [68, 69]. In our investigation, moderate treadmill physical exercise was able to modulate enzymatic and nonenzymatic antioxidant parameters in the cerebellum, which has led to the maintenance of T-AOC and avoided oxidative stress by increasing lipid peroxidation. A study on the effect of low-intensity aerobic exercise in humans after 12 and 24 months reported that aerobic exercise was able to modulate antioxidant enzymes; however, this seems to be time dependent since only a difference in superoxide dismutase (SOD) activity was observed at 12 months, while differences in catalase activity (CAT) and reduction of blood lipid peroxidation were found after 24 months of exercise [69]. However, the effect of exercise on activities of antioxidant enzymes is still conflicting. Some authors report that the effects of exercise on antioxidant enzyme activities are dependent on the brain region, in certain parts such as the stem and corpus striatum; physical exercise resulted in increased activities of superoxide dismutase (SOD) and GPX [70, 71], while other brain areas do not change, as in the cerebellum. Another important aspect is that PE by treadmill running also did not alter the activities of SOD, catalase, or GPX in the rat brain [66]. In general, it is suggested that physical exercise selectively regulates the antioxidant factors, mainly enzymatic, in different regions of the brain, which is probably dependent on the type of physical activity, intensity and duration of physical exercise, age, sex, and strain of rats [66].

In an animal study, a 6-week aerobic training protocol of moderate intensity did not result in SOD difference; however, CAT alterations and oxidative stress reduction in regions associated with motor functions such as motor cortex, cerebellum, and striatum were observed [68]. No improvement was noted by using our protocol comprised of 4 weeks of aerobic resistance exercise with moderate intensity [18]; however, the antioxidant parameters were not altered in comparison to the control group, which indicates a protective effect on the antioxidant activity [68]. Exercise was not able to improve the antioxidant system capacity of animals that did not consume EtOH; this can be explained by the fact that antioxidant responses depend on the exercise type, volume, and intensity of the training protocol [72].

Although the antioxidant parameters were not improved, the exercise protocol was able to avoid the reduction of myelin basic protein (MBP) immunolabeling, as well as the decrease of Purkinje cell density caused by the EtOH consumption. These results may arise from the ability of moderate aerobic exercise to increase the expression of neurotrophic factors such as BDNF (brain-derived neurotrophic factor), FGF (fibroblast growth factor), and VEGF (vascular endothelial growth factor), which are responsible for development and maintenance of the nervous system [6, 73]. The physical exercise has modulatory effects on energy hemostasis through improving mitochondrial function, leading to increased levels of BDNF that activates the transcription factor cAMP response element-binding protein (CREB) through tyrosine-related kinase B (TrkB) receptors. This process creates a positive cycle for the cascades, as well as induces autophagy and promotes the antioxidant system, making the neurons more resistant to oxidative stress by activating regulatory mechanisms in that cell [74]. DNA-binding sites for CREB contribute the increase in the expression of BDNF mRNA, a process that can be regulated by ROS; thus, the increase of ROS stimulates the expression of these factors that play a key role in the control of the oxidative process [66, 75]. Therefore, exercise can lead to redox homeostasis by stimulating the regulatory process.

In this regard, regular physical exercise attenuated the accumulation of carbonylated proteins related to oxidative stress in the brain associated with age, due to increased proteasome activity and thus improved brain function [76]. Studies with transgenic mice that accumulate beta-amyloid proteins have also shown that voluntary exercise decreased beta-amyloid accumulation in the brain by increasing the activity of neprilysin, a protein responsible for the degradation of beta-amyloids [77]. Thereby, biochemical changes can lead to cellular damage and result in motor activity reduction, as seen in this study. In contrast, physical exercise was able to protect the CNS from all changes caused by EtOH, including biochemical, cellular, and behavioral damages. This entire protection is conditioned to the exercise capacity to modulate the antioxidant process, growth factors, and maintenance of CNS [6, 73].

## 5. Conclusions

**Figure 10:**
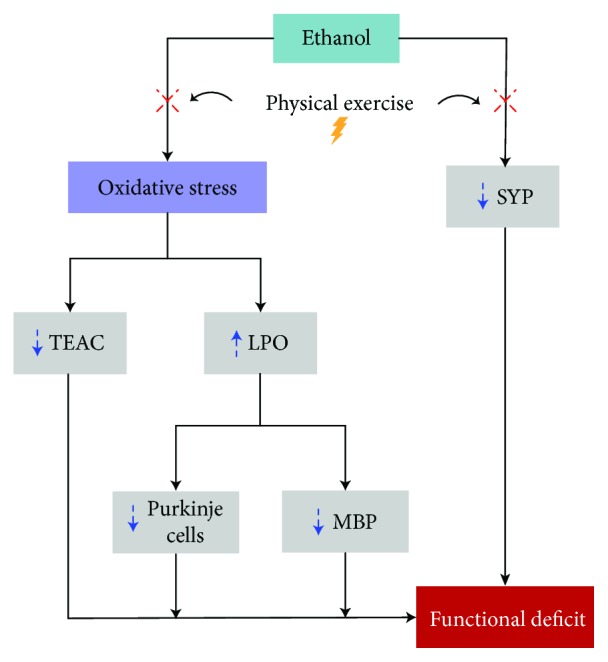
Description of the main results found in this article. Exposure to binge-like ethanol caused lower antisynaptophysin (SYP) immunostaining and oxidative stress, from the decrease of Trolox equivalent antioxidant capacity (TEAC) levels and higher lipid peroxidation (LPO). The oxidative biochemistry misbalance induced tissue damages, as decrease of Purkinje cell population and antimyelin basic protein (MBP) immunostaining. The oxidative biochemistry and tissue damage were the main factors responsible for fine motor control changes. In addition, we indicated the role of physical exercise in damage ways in exposure to binge-like ethanol.

We demonstrated that that EtOH in binge drinking induces oxidative biochemistry imbalance, from the decrease of TEAC levels and higher LPO production, which can trigger tissue and behavioral damages in rats. Finally, the decrease in the number of cells was probably the main factor responsible for changes on fine motor control—the main findings of this paper are summarized in Figure 10. One of the main contributions of the present study was to show for the first time that the damages promoted in the cerebellum of rats by EtOH binge drinking can be minimized by moderate treadmill physical exercise, avoiding the oxidative imbalance and minimizing the tissue and functional damages in the cerebellum caused by ethanol, possibly leading to redox homeostasis by stimulating the regulatory process.

## Data Availability

The quantitative and qualitative data used to support the findings of this study are included within the article.
